# A Tailored Advice Tool to Prevent Injuries Among Novice Runners: Protocol for a Randomized Controlled Trial

**DOI:** 10.2196/resprot.9708

**Published:** 2018-12-19

**Authors:** Ellen Kemler, Vincent Gouttebarge

**Affiliations:** 1 Dutch Consumer Safety Institute Amsterdam Netherlands; 2 Department of Orthopaedic Surgery Amsterdam University Medical Centers, University of Amsterdam Amsterdam Movement Sciences Amsterdam Netherlands; 3 Division of Exercise Science and Sports Medicine University of Cape Town Cape Town South Africa; 4 Amsterdam Collaboration on Health & Safety in Sports AMC/VUmc IOC Research Center Amsterdam Netherlands

**Keywords:** behavior, injury, prevention, running

## Abstract

**Background:**

Besides the beneficial health effects of being active, running is associated with a risk of sustaining injuries. Runners need to change their behavior to increase the use of effective measures and subsequently reduce the number of running-related injuries.

**Objective:**

The RunFitCheck intervention was developed according to an evidence- and practice-based approach to stimulate injury-preventive behavior among novice runners. This paper describes the study design in detail.

**Methods:**

A randomized controlled trial with a follow-up period of 5 months will be conducted. The participants will be novice runners. At enrollment, participants will be asked to report injury-preventive measures they usually take during their running activities. After completing the enrollment questionnaire, participants will be randomized to intervention and control groups. The intervention group will have access to the RunFitCheck intervention; the control group will perform their running activities as usual. Participants will be asked to report retrospectively in detail what they have done regarding injury prevention during their running activities at 1, 3, and 5 months after enrollment. Descriptive analyses will be conducted for different baseline variables in the intervention and control group. Relative risks and 95% CIs will be used to analyze behavioral changes according to the intention-to-treat principle.

**Results:**

The project was funded in 2016 and enrollment was completed in 2017. Data analysis is currently under way and the results are expected to be submitted for publication in 2019.

**Conclusions:**

To nullify the negative side effects of running, prevention of training errors is desirable. As the use of injury prevention measures is not compulsory in running, a behavioral change is necessary to increase the use of effective injury-preventive measures and to prevent running-related injuries.

**Trial Registration:**

Netherlands Trial Register NTR6381; http://www.trialregister.nl/trialreg/admin/rctview.asp?TC=6381 (Archived by WebCite at http://www.webcitation.org/736Xjm5jv)

**International Registered Report Identifier (IRRID):**

RR1-10.2196/9708

## Introduction

Running is one of the most popular ways to exercise in the Netherlands, with 2.1 million people running on a regular basis [1]. In addition to beneficial health effects of being active, running is associated with a high risk of musculoskeletal injuries. The incidence of running-related injuries (RRIs) is reported to range from 3-59 injuries per 1000 exposure hours [2-4], with most RRIs occurring in the knee, followed by the calf, Achilles tendon, and shin [5].

Risk factors for RRIs have been extensively investigated, but evidence remains contradictory and inconclusive. A history of injury in the past 12 months is reported to be the main risk factor for RRIs [6,7]. Especially, novice runners are at a high risk of sustaining an RRI, while according to running experts, half of the RRIs are related to training errors [3]. Consequently, these injuries could be prevented since modifying the training load should be easily realizable [8,9]. However, modifying one’s training load and inherently preventing training errors might be easier said than done. A behavioral change among runners is necessary to increase injury-preventive behavior and, subsequently, the use of preventive measures. To our knowledge, 2 interventions aimed at runners to promote preventive behavior and reduce RRIs have been developed earlier [10,11]. Adriaensens et al [10] developed a tailored Web-based injury prevention intervention: a website with informational videos about the etiology and mechanisms of RRIs combined with injury-preventive advice and a Web-based questionnaire that allowed the website to provide tailored feedback based upon a series of predefined questions that created a personal risk profile of the user. Short-term (3 months) effects of the intervention (measured in recreational runners after a visit to the website of 30 minutes) were demonstrated on determinants and actual performance of sports injury behavior. Hespanhol et al [11] evaluated the effectiveness of adding Web-based tailored advice to general advice on the prevention of RRIs and determinants as well as actual preventive behavior in Dutch trail runners. Trail runners in the intervention group received specific advice tailored to their RRI status (no injury, no substantial RRI, or substantial RRI). In that study, no effect was observed on determinant and actual preventive behavior; however, RRIs were prevented [11]. Tailored Web-based intervention can thus be promising in promoting preventive behavior among runners and even prevent RRIs. The intervention developed by Adriaensens et al (Dutch Consumer Safety Institute) [10] was effective, but the Web-based questionnaire for tailored feedback was time-consuming. Therefore, the Dutch Consumer Safety Institute developed the RunFitCheck intervention to stimulate injury-preventive behavior among novice runners without the associated time burden. The next step is to evaluate its effect on injury-preventive behavior among novice runners. This paper describes the study design in detail.

## Methods

### Objective and Hypothesis

The objective of the study is to evaluate the effectiveness of the developed RunFitCheck intervention (in Dutch) to promote injury-preventive behavior among novice runners. The hypothesis in this study is that the developed intervention will lead to 10% difference in favorable injury-preventive behavior in the intervention group compared with the control group.

### Study Design

**Figure 1:**
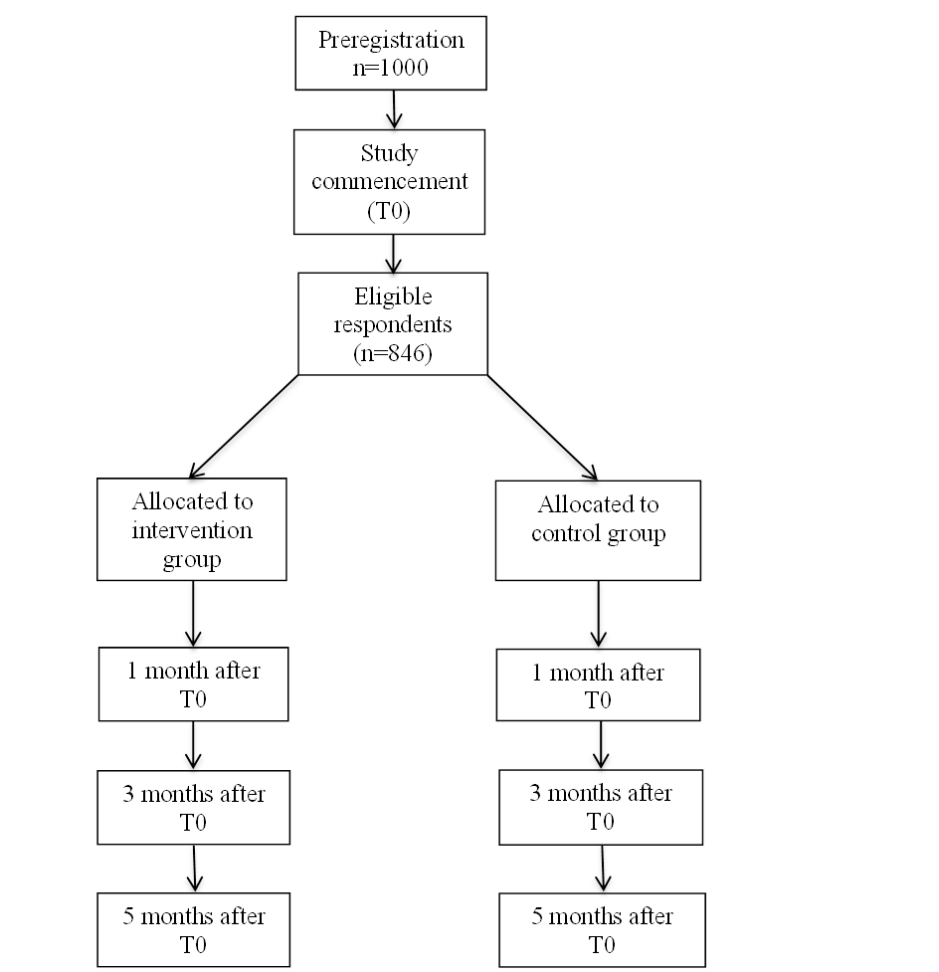
Flowchart of participants of the randomized prospective controlled trial.

The Consolidated Standards of Reporting Trials statement was followed to describe the design of the study [8]. This statement is a checklist intended to improve the quality of reports of randomized controlled trials. A randomized controlled trial with a follow-up period of 5 months will be conducted (Figure 1). The study is registered in the Dutch Trial Registry (Netherlands Trial Register Number NTR6381).

### Ethics, Consent, and Permissions

The study protocol has been approved by the Medical Ethics Review Committee of the Academic Medical Center Amsterdam, the Netherlands (W16_335 # 16.417). Participants willing to participate in the study will give their informed consent prior to the start of the enrollment questionnaire. The start screen of the enrollment questionnaire will be used to obtain informed consent from the participants. This screen will contain information about the study, use of the data gathered, and rights of the participants in this study. If participants agree to participate in this study after they have read the information, they will have to tick a box to give their informed consent. When participants tick the informed consent box, they are able to continue to the first question of the enrollment questionnaire. The flowchart of the participants is presented in Figure 1.

### Participants and Recruitment

The group of participants will consist of novice runners. The inclusion criteria are being aged 18 years and older, considering oneself as a beginner, a slightly experienced runner, or a rather experienced runner, and having less than one year of running experience when a runner considers oneself as an experienced or very experienced runner. Participants do not have to be a member of a running club. Participants will be recruited via social media networks (Facebook, websites, Twitter, LinkedIn, and newsletters) of the participating organizations (Dutch Consumer Safety Institute, Runner’s World, and Royal Dutch Running Association [RDRA]) and will be able to preregister for the study in the 2 months prior to the start of the study.

### Sample Size and Allocation

In this study, the aim is to increase injury-preventive behavior by 10% in the intervention group. In previous literature, 12.6% increase in injury-preventive behavior (in this case, the inclusion of a warm-up) was found during a 3-month intervention [10]. To achieve 80% power with a significance level of.05, the sample size calculation revealed that 384 participants per study group are needed in this study. Taking into account a response rate of 85% and a dropout rate of 10% over the 5-month follow-up period, a total of 1000 participants will be approached in this study. Eligible participants will be simultaneously allocated at random to the intervention or control group after study commencement, using a computerized random number generator (the Aselect function in Excel). No restrictions will be imposed for the allocation; simple randomization will be performed. All steps in the randomization process will be done by the principal researcher.

### Intervention

The RunFitCheck intervention (in Dutch), is an intervention developed according to an evidence-based (intervention mapping) and practice-based (running experts) approach to stimulate injury-preventive behavior among novice runners (see Multimedia Appendices 1, 2, and 3). The development of the intervention was a collaboration with running experts and the target group and is described in detail in Kemler, Valkenberg, and Gouttebarge (in press).

A range of experts was consulted in the development of this intervention: a sports physician who is head of the medical commission of the RDRA and related to the RDRA as a sports physician since 1977; a sports physiotherapist specialized in running since 1992; 2 running coaches, of whom 1 is a fulltime running coach since 2002 and coach of both successful Dutch top athletes and recreational athletes, and a second coach who has over 40 years of running experience, was a top athlete himself, coaches recreational athletes, and has worked for the RDRA for 20 years; and a researcher specialized in sports injuries who performed the running-related literature review that laid the foundations for this study. These experts were consulted and their feedback combined with literature on the use of running apps (Romeyn, Kemler and Huisstede, in press). Through the consultation and research process, 2 main dimensions in the risk for RRIs, namely the physical load-taking capacity of runners and motivation of runners to achieve their running goals, were identified. Across these 2 domains, runners can be classified in 4 categories: (1) a low physical load-taking capacity and a low goal-orientation, (2) a low physical load-taking capacity and a high goal-orientation, (3) a high physical load-taking capacity and a low goal-orientation, and (4) a high physical load-taking capacity and a high goal-orientation. In order to classify runners within 1 of the 4 categories, they will answer 6 questions (Multimedia Appendix 4). Depending on their answers and related classification, runners will directly receive tailored advice on the website for achieving an optimal running practice. Examples of advice will be related to strength exercises, running technique improvement, and use of a training schedule. Advice about a warm-up will also be given. After receiving the tailored advice, runners can leave their email address on the website so that they will receive a tailored running schedule and a strength exercise schedule every week. The RunFitCheck intervention will be made available to the participants within the intervention group. Substantial efforts have been made to develop an intervention that provides practical injury prevention advice.

### Running as Usual

The participants in the control group will perform their running activities as usual. The RunFitCheck website is not secured with a log-in code for the intervention group as the research team wants to avoid any additional barriers for participants to use the intervention. Participants in the control group will be able to visit and use the website alongside those in the intervention group; however, they will not be actively encouraged to do so during the study period. In order to determine whether control group participants accessed RunFitCheck, data from control group participants pertaining to websites they visit during the study period will be collected. Control group participants who have visited RunFitCheck will be excluded from the analyses.

### Injury-Preventive Behavior

As the intervention developed by Adriaensens et al (Dutch Consumer Safety Institute) [10] was effective in promoting injury-preventive behavior but was time-consuming, the research team wanted to develop an intervention to stimulate injury-preventive behavior among novice runners without the associated time burden. In this study, injury-preventive behavior is defined as follows: (1) using a (personalized) training schedule: use of a training schedule that fits the physical condition of the athlete and represents a realistic running goal was proposed by running experts as an important factor in preventing running injuries; (2) performing strength and technique exercises [12] to improve the amount of force that can be exerted on the body while running; and (3) performing an active warm-up prior to running [13]. Each of these injury-preventive behaviors is divided into preparatory acts, such as searching for information about strength exercises and structural injury-preventive measures like performing strength exercises. All injury-preventive behaviors are assessed through single questions (yes, no, or not applicable).

### Procedures

Upon enrollment, participants will be asked to report the injury-preventive measures they usually take before or during their running activities. Participants’ demographic characteristics, including their contact email address, sex, age, running experience, running motivation, physical fitness, current injuries and physical complaints, and injury-preventive measures, will also be collected upon enrollment via a Web-based enrollment questionnaire (Table 1).

**Table 1:** Overview of research topics per questionnaire in this study.

Research topics	Enrollment questionnaire	At 1 month after enrollment	At 3 months after enrollment	At 5 months after enrollment
Age	Present	N/A^a^	N/A	N/A
Sex	Present	N/A	N/A	N/A
Sports participation other than running	Present	Absent	Absent	Absent
Running motivation	Present	Absent	Absent	Absent
Running experience (in months or years)	Present	Absent	Absent	Absent
Level of running (novice, seminovice, experienced, etc)	Present	Absent	Absent	Absent
Running frequency per week	Present	Present	Present	Present
Physical fitness	Present	Present	Present	Present
Risk perception	Present	Present	Present	Present
Injury-preventive behavior	Present	Present	Present	Present
Running-related injuries	Present	Present	Present	Present
Physical complaints	Present	Present	Present	Present

^a^N/A: not applicable.

All personal information and contact information from the participants will be handled by the principal researcher. This researcher will export the data from SurveyMonkey and store it in a password-protected drive. Participant names will be replaced with numbers and stored as reidentifiable data. The data with participant numbers will be utilized in the statistical analyses. Upon the conclusion of the data collection period, the questionnaire and associated responses will be deleted from SurveyMonkey.

Questionnaires will be administered at study commencement and at 1, 3, and 5 months post-commencement. In these follow-up questionnaires, participants will be asked to retrospectively report in detail what they have done in the past month(s) regarding injury prevention during their running activities and will be asked questions about their current injury-preventive behavior, running activities, and running injuries during running activities. The injury definition utilized in this study is “any physical complaint arising during a running activity that does not allow the participant to complete the activity or affects their ability to participate in future running activity” [14-17].

At 4 time points, a Web-based questionnaire will be sent by email to participants by the principal researcher, using the Web-based questionnaire system SurveyMonkey. When participants fail to complete a questionnaire, reminder notifications will be sent via email by the principal researcher using the Web-based questionnaire system SurveyMonkey 7 days after a questionnaire is due. A second reminder will be sent after 2 weeks.

The evaluation of the effectiveness of RunFitCheck will not take place in an experimental setting. Participants in the intervention group will be given access to the intervention, but no further conditions will be applied to the use of the intervention. In the questionnaires at 1, 3, and 5 months, the intervention group will be asked about their use of the intervention, attractiveness of the intervention, behavioral actions they take after being exposed to the intervention, their intention to use this intervention again, and how they would want to be informed about this intervention. If participants indicate they do not use the intervention, they will be asked to give detailed information about why they do not use the intervention.

### Statistical Analysis

Descriptive analyses (mean [SD], frequency, and range) will be conducted for the different baseline variables in both study groups. To evaluate the success of the randomization, baseline variables will be analyzed for differences between the intervention and control group (chi-square test, independent *t* test, and Mann-Whitney test).

Relative risks (RRs) and a 95% CI will be used to analyze behavioral change according to the intention-to-treat analyses: (1) using a (personalized) training schedule, (2) performing strength and technique exercises, (3) performing a warm-up. Participants who do not execute a certain injury-preventive measure at study commencement will be selected for analysis, since among these participants, most behavioral change can be expected. If there are any differences in descriptive characteristics at baseline (eg, differences in sex, age, or running experience), subanalyses for sex, different age categories, etc will be performed.

## Results

The project was funded in 2016 and enrollment was completed in 2017. Data analysis is currently under way and the results are expected to be submitted for publication in 2019.

## Discussion

### Principal Findings

This article describes the design of a randomized controlled trial evaluating the effectiveness of an intervention on injury-preventive behavior in novice runners. There are 3 major challenges in this study that can be acknowledged: (1) the recruitment of participants, (2) their adherence to this study, and (3) the measurement of injury-preventive behavior.

### Recruitment of Participants

For the study results to be meaningful, 1000 novice runners need to be enrolled. To enhance enrollment and the possibility to preregister for the study, several strategies will be used, such as social media (Facebook, LinkedIn, and Twitter), newsletters from the RDRA (digital), and the magazine Runner’s World (in print and digital). Preregistration will be possible by filling in a Web-based form on the website of the Dutch Consumer Safety Institute. Preregistration for the study will be advertised on Facebook and Twitter.

Social media has been previously utilized by the research team to recruit runners with close to 1000 participants recruited within 2 weeks. Respondents were recruited via social media, such as Facebook and Twitter, and they were invited to give their opinion on running, running injuries, and injury prevention. As the research team recruited nearly 1000 runners within 2 weeks, they are confident that they will achieve 1000 participants in this study with similar recruitment strategies.

### Adherence to the Study

To provide an incentive to complete all components of the study, enrolled participants who complete all questionnaires will be entered into a draw to win a €200 gift voucher for running clothes. This strategy might enhance the respondents’ adherence to the study. The gift voucher for running clothes will be a gift from RDRA. RDRA supports the RunFitCheck intervention but is not otherwise related to this study.

### Injury-Preventive Behavior

As the intervention developed by Adriaensens et al (Dutch Consumer Safety Institute) [10] was effective in promoting injury-preventive behavior but time-consuming, the Dutch Consumer Safety Institute wants to develop an intervention to stimulate injury-preventive behavior among novice runners without the associated time burden. Therefore, injury-preventive behavior as a primary outcome in our study instead of the more traditional RRIs was chosen. It is well known that people find it difficult to change health-related behavior. Sustaining lasting change in health-related behavior is a detailed and lengthy process [18]. Our study assesses a small part of this process, focusing on a 10% change in injury-preventive behavior as an indication that the intervention is efficacious.

## Appendix

Multimedia Appendix 1 Measuring goal-orientation of runners.

Multimedia Appendix 2 Classification of the physical load-taking capacity of runners and their motivation with regard to achieving their running goals.

Multimedia Appendix 3 Advice for strength exercises.

Multimedia Appendix 4 Short questionnaire to classify runners.

## Abbreviations

RDRA: Royal Dutch Running Association
RR: relative risks
RRI: running-related injuries

## References

[1] (2014). VeiligheidNL.

[2] van Gent RN, Siem D, van Middelkoop M, van Os AG, Bierma-Zeinstra SMA, Koes BW (2007). Incidence and determinants of lower extremity running injuries in long distance runners: a systematic review. Br J Sports Med.

[3] Buist I, Bredeweg SW, Bessem B, van Mechelen W, Lemmink KAPM, Diercks RL (2010). Incidence and risk factors of running-related injuries during preparation for a 4-mile recreational running event. Br J Sports Med.

[4] Kluitenberg B, van Middelkoop Marienke, Verhagen E, Hartgens F, Huisstede B, Diercks R, van der Worp Henk (2016). The impact of injury definition on injury surveillance in novice runners. J Sci Med Sport.

[5] Kluitenberg B, van Middelkoop M, Smits DW, Verhagen E, Hartgens F, Diercks R, van der Worp H (2015). The NLstart2run study: Incidence and risk factors of running-related injuries in novice runners. Scand J Med Sci Sports.

[6] Saragiotto BT, Yamato TP, Hespanhol Junior Luiz Carlos, Rainbow MJ, Davis IS, Lopes AD (2014). What are the main risk factors for running-related injuries?. Sports Med.

[7] Hulme A, Nielsen RO, Timpka T, Verhagen E, Finch C (2017). Risk and Protective Factors for Middle- and Long-Distance Running-Related Injury. Sports Med.

[8] Hreljac A (2005). Etiology, prevention, and early intervention of overuse injuries in runners: a biomechanical perspective. Phys Med Rehabil Clin N Am.

[9] Fields KB, Sykes JC, Walker KM, Jackson JC (2010). Prevention of running injuries. Curr Sports Med Rep.

[10] Adriaensens L, Hesselink A, Fabrie M, Brugmans M, Verhagen E (2014). Effectiveness of an tailored intervention on determinants and behavior to prevent running related sports injuries: a randomized controlled trial. Schweizerische Zeitschrift f&uuml;r Sportsmedizin und Sporttraummatologie.

[11] Hespanhol Luiz Carlos, van Mechelen Willem, Verhagen E (2018). Effectiveness of online tailored advice to prevent running-related injuries and promote preventive behaviour in Dutch trail runners: a pragmatic randomised controlled trial. Br J Sports Med.

[12] Niemuth PE, Johnson RJ, Myers MJ, Thieman TJ (2005). Hip muscle weakness and overuse injuries in recreational runners. Clin J Sport Med.

[13] Behm DG, Blazevich AJ, Kay AD, McHugh M (2016). Acute effects of muscle stretching on physical performance, range of motion, and injury incidence in healthy active individuals: a systematic review. Appl Physiol Nutr Metab.

[14] Fuller CW, Ekstrand J, Junge A, Andersen TE, Bahr R, Dvorak J, H&auml;gglund M, McCrory P, Meeuwisse WH (2006). Consensus statement on injury definitions and data collection procedures in studies of football (soccer) injuries. Br J Sports Med.

[15] Fuller CW, Molloy MG, Bagate C, Bahr R, Brooks JHM, Donson H, Kemp SPT, McCrory P, McIntosh AS, Meeuwisse WH, Quarrie KL, Raftery M, Wiley P (2007). Consensus statement on injury definitions and data collection procedures for studies of injuries in rugby union. Br J Sports Med.

[16] Pluim BM, Fuller CW, Batt ME, Chase L, Hainline B, Miller S, Montalvan B, Renstr&ouml;m Per, Stroia KA, Weber K, Wood TO, Tennis Consensus Group (2009). Consensus statement on epidemiological studies of medical conditions in tennis, April 2009. Clin J Sport Med.

[17] Bahr R (2009). No injuries, but plenty of pain? On the methodology for recording overuse symptoms in sports. Br J Sports Med.

[18] Kelly MP, Barker M (2016). Why is changing health-related behaviour so difficult?. Public Health.

