# Supplementary figures and images for: A Tailored Advice Tool to Prevent Injuries Among Novice Runners: Protocol for a Randomized Controlled Trial
Source: JMIR Res Protoc. 2018 Dec 19;7(12):e187. doi: 10.2196/resprot.9708 (PMC6315232; doi:10.2196/resprot.9708)

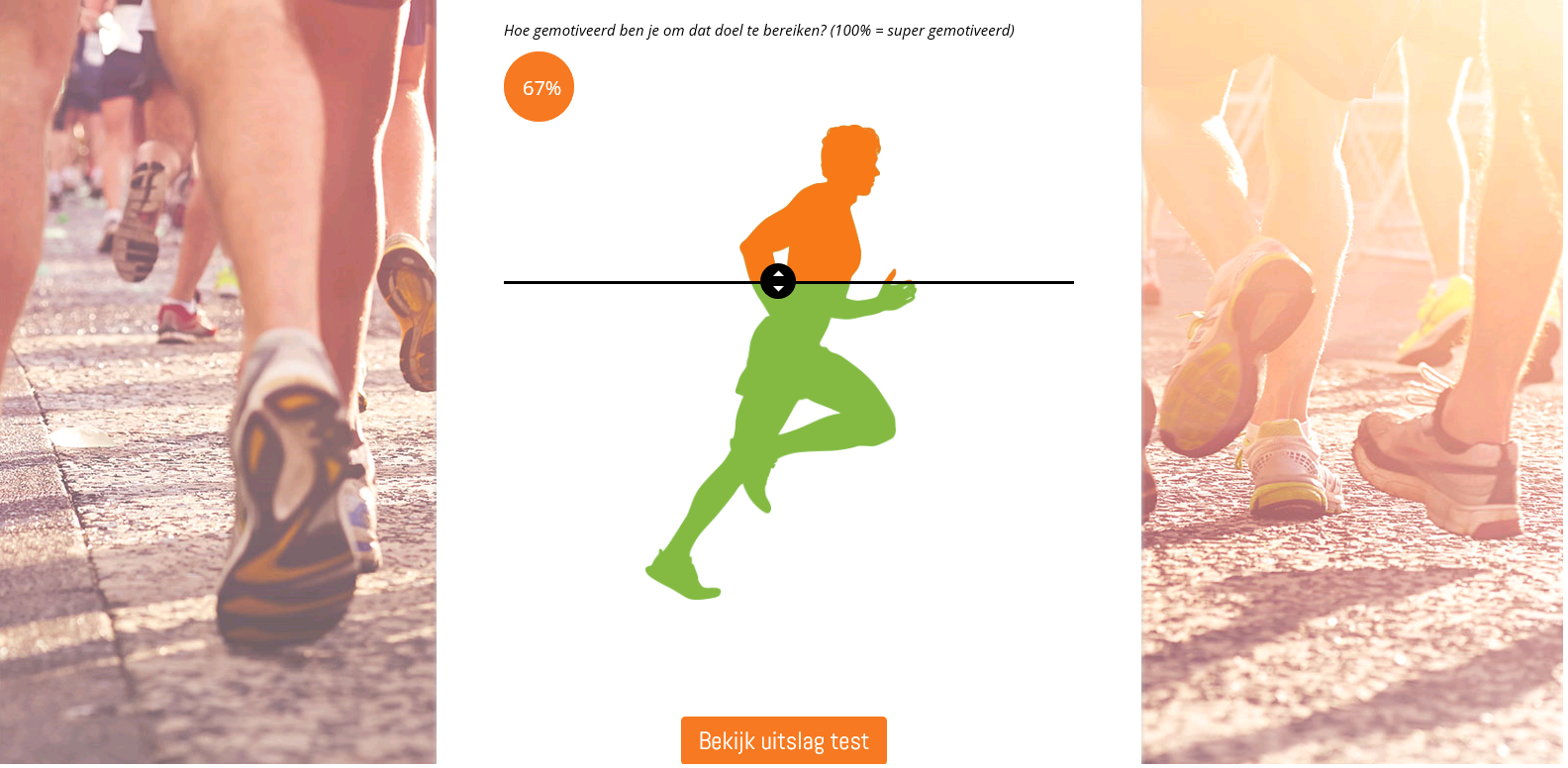

Supplement: Multimedia Appendix 1 [file resprot_v7i12e187_app1.png]

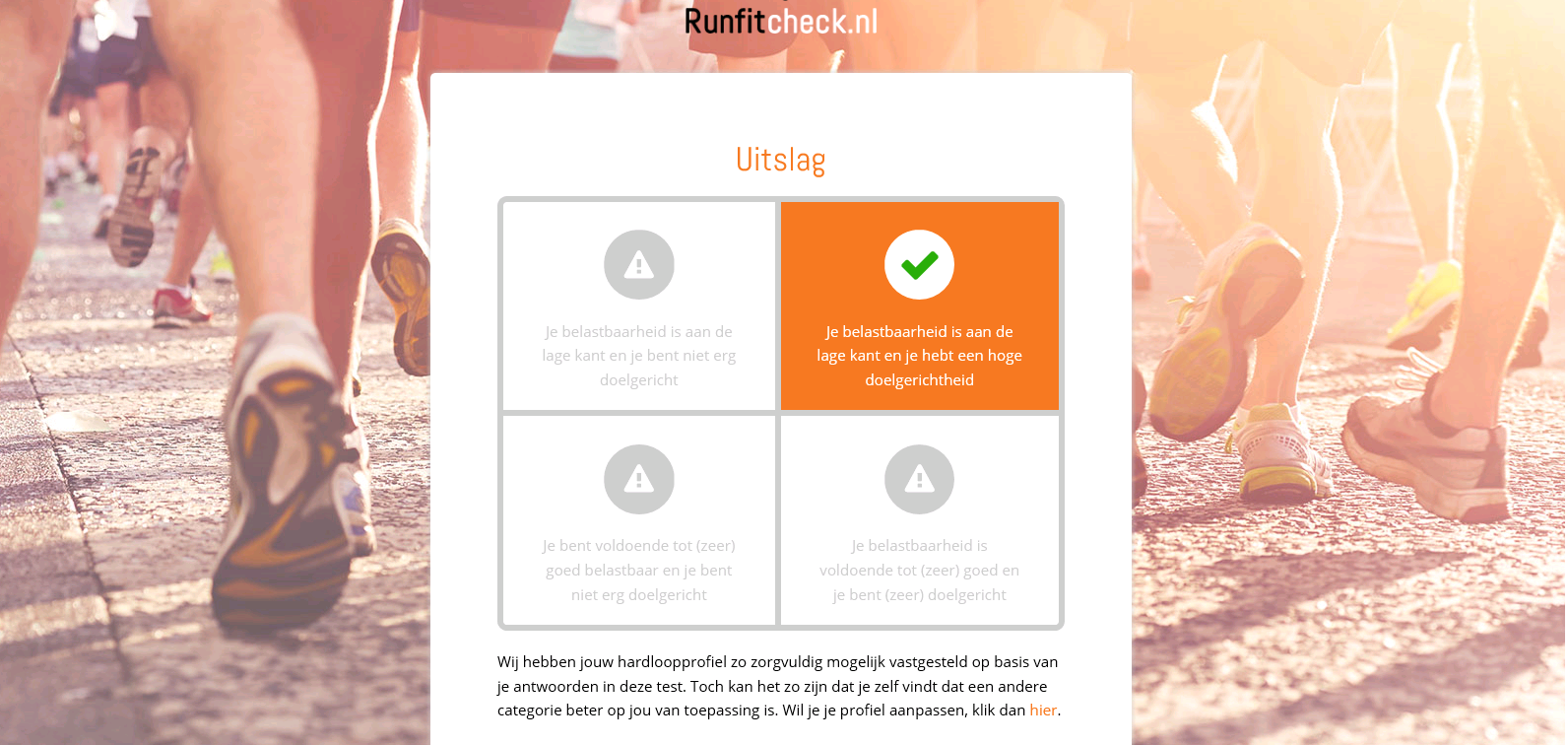

Supplement: Multimedia Appendix 2 [file resprot_v7i12e187_app2.png]

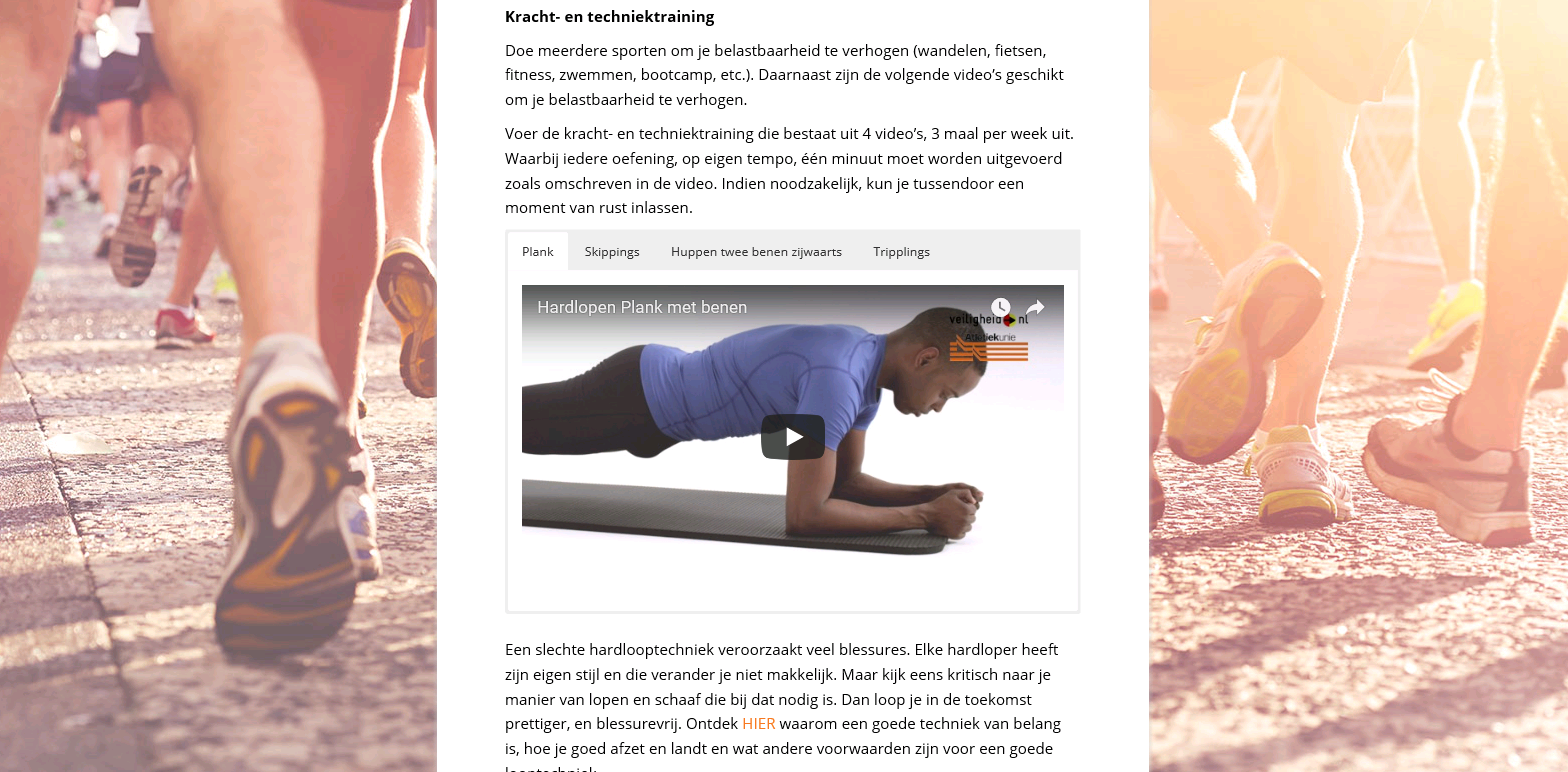

Supplement: Multimedia Appendix 3 [file resprot_v7i12e187_app3.png]
