# Supplementary material for: A Tailored Advice Tool to Prevent Injuries Among Novice Runners: Protocol for a Randomized Controlled Trial
Source: JMIR Res Protoc. 2018 Dec 19;7(12):e187. doi: 10.2196/resprot.9708 (PMC6315232; doi:10.2196/resprot.9708)
Supplement: Multimedia Appendix 4 [file resprot_v7i12e187_app4.pdf]

Appendix D Short questionnaire for runners

- How many times a week - on average - did you run in the past 3 months?
- How many times a week - on average - did you exercise apart from running in the last 3 months?
- What score would you assign to your general endurance? (How long can you continue to perform a specific effort) *Scale 1 – 10+*
- How sensitive are you to injuries? *Scale 1 – 10+*
- What is your main goal in running?

  ☐ Training for a running event

  ☐ Training to be able to run a certain distance

  ☐ Improving endurance, fitness

  ☐ Enjoying exercise, social interaction, exercise in a group

  ☐ Lose weight

  ☐ Clear my head
- How motivated are you to reach that goal? (0 – 100%, 100% = highly motivated)
